# In-Situ Single-Particle Sensing of Carbonaceous Aerosols in Freshly Generated Smoke Using Optical Trapping–Raman Spectroscopy

**DOI:** 10.3390/s26206527

**Published:** 2026-10-15

**Authors:** Yukai Ai, Chuji Wang, Yong-Le Pan, Gorden Videen

**Affiliations:** 1 Department of Physics and Astronomy, Mississippi State University, Starkville, MS 39759, USA; yya7@msstate.edu; 2 Space Science Institute, 1601 29th St. Suite 1292, Boulder, CO 80301, USA; 3 DEVCOM Army Research Laboratory, 2800 Powder Mill Road, Adelphi, MD 20783, USA; 4 Humanitas College, Kyung Hee University, 1732, Deogyeong-daero, Giheung-gu, Yongin-si 17104, Gyeonggi-do, Republic of Korea

**Keywords:** in-situ sensing, carbonaceous aerosols, single-particle, optical trapping, Raman spectroscopy, spatial resolution

## Abstract

**Highlights:**

**What are the main findings?**

- In-situ, contact-free sensing of the native properties of single airborne particles.
- High spatial resolution reveals chemical heterogeneity within a single particle.

**What is the implication of the main finding?**

- These capabilities support future on-site sensing of single airborne aerosols.

**Abstract:**

Carbonaceous aerosols represent a major component of atmospheric particulate matter. However, current characterization methods have limitations in sensing these particles because they either measure only one type of aerosol property or rely on offline measurement from collected samples. In-situ sensing is crucial for characterizing the physical and chemical properties of carbonaceous aerosols while they remain airborne. In this work, optical trapping–Raman spectroscopy (OT–RS) was applied for in-situ sensing of single carbonaceous aerosol particles in freshly generated smoke. Nylon rope and plastic fiber were ignited inside an open chamber using the trapping laser itself. The resulting particles were captured directly in air by the optical trap and immediately analyzed using Raman spectroscopy and real-time imaging within seconds. The optically trapped particles had spatial dimensions of several micrometers and estimated masses on the order of ~10 pg. We demonstrate that this minute amount of material is sufficient to obtain detailed Raman spectral fingerprints. Furthermore, analysis of the Raman bands allowed two types of particles to be clearly distinguished based on their distinct Raman features. Spatially resolved Raman spectra further resolved that individual trapped particles are chemically heterogeneous, with organic carbon and soot located in different regions within the same particle. These results demonstrate that OT–RS provides an effective platform for in-situ characterization of single carbonaceous aerosol particles and possesses strong potential for future on-site atmospheric sensing and monitoring.

## 1. Introduction

Carbonaceous aerosols refer to the carbon-containing fraction of atmospheric particulate matter. Based on differences in composition, carbonaceous aerosols are broadly divided into organic carbon (OC) and elemental carbon (EC) [1,2,3,4]. OC includes directly emitted primary organic aerosols and secondary organic aerosols formed through atmospheric reactions, whereas EC represents a thermally refractory, graphitic-like carbon fraction [2]. Alternatively, they can also be classified by their light-absorption properties into black carbon (BC) and brown carbon (BrC) [5,6,7,8,9,10]. BC absorbs strongly across the visible spectrum with a relatively weak wavelength dependence and is primarily associated with refractory, soot-like carbon [5,6]. In contrast, BrC consists mainly of light-absorbing organic carbon with absorption that increases toward shorter visible and ultraviolet wavelengths [5,7]. This wavelength-dependent absorption originates from diverse organic chromophores and varies depending on the emission source and atmospheric aging processes [7].

Combustion contributes most of the carbonaceous aerosols in the atmosphere [10]. Burning fossil fuels, biofuels, biomass, industrial materials, and waste releases soot, polycyclic aromatic hydrocarbons (PAHs), and other organic carbonaceous aerosols [1,10]. Wildfires represent a major source of combustion-generated carbonaceous aerosol [10,11]. Recent Canadian wildfire seasons show that carbonaceous aerosols within smoke can travel far from their source and affect large regions [12]. Freshly generated smoke particles are also highly dynamic. Following emission, their physical and chemical properties can evolve via oxidation, condensation, evaporation, coagulation, and heterogeneous reactions. These processes can alter particle composition, morphology, mixing state, and surface properties. Therefore, in-situ characterization of freshly generated airborne smoke particles is crucial to understand the initial properties of these aerosols and their subsequent atmospheric evolution. Overall, carbonaceous aerosols are not uniform materials but a heterogeneous population of particles with different carbon-bonding structures, functional groups, coatings, sizes, morphologies, and internal or external mixing states, and can be transported over long distances and affect large areas [13].

Methods for sensing and characterizing carbonaceous aerosols can be broadly categorized into physical and chemical analyses. Physical measurements determine particle number, size, mass, morphology, and optical properties. Condensation particle counters measure particle number [14], while elastic scattering, mobility and aerodynamic particle sizers measure size distributions [14,15]. Filter weighing determines total particle mass, and microscopy provides detailed particle size and morphology [14]. Aethalometers and photoacoustic instruments characterize light absorption, whereas laser-induced incandescence quantifies refractory black carbon [16]. Chemical analyses elucidate molecular or elemental composition. Thermal–optical analysis separates OC from EC [2], and mass spectrometry identifies elemental composition [17]. Spectroscopic techniques can resolve chemical composition; for example, Raman spectroscopy provides molecular fingerprints of the targeted aerosol samples [18].

Despite the availability of these methodologies, several critical limitations persist. First, most approaches quantify only a single particle property, failing to provide a complete physical and chemical description. For example, light-absorption instruments measure wavelength-dependent optical signals that can be interpreted with models to estimate BC- and BrC-related fractions; however, they cannot directly classify these materials or identify their molecular compositions [2,5,16]. Second, many methods require a sampling process; particles have to be collected and measured on substrates [19,20]. The collection process and substrate alter the particle properties and introduce contamination and background interference [19,20]. Third, many detailed chemical and structural measurement methods provide only offline measurements [18,20]. The delay between particle emission, collection, and analysis allows the sample to undergo unwanted physical and chemical transformations [18,20]. During this period, particles may react with atmospheric gases, undergo hygroscopic growth, oxidize, volatilize, or coagulate with neighboring particles. As a result, the measured spectra may represent an aged particle rather than its native airborne state. Therefore, in-situ, contact-free sensing that simultaneously captures both the physical and chemical properties of airborne aerosol particles within a single system is needed for characterizing them in their native state.

Single-particle measurement offers a robust approach for characterizing airborne aerosols [13,18,20,21,22]. This technique evaluates individual particles rather than averaging data across a bulk population, thereby revealing distinct variations in composition, size, phase, and morphology [13]. Furthermore, it requires minimal sample volumes. Previous studies have demonstrated detection at subpicogram-to-picogram mass scales [23,24]. For instance, Vehring et al. [23] reported a Raman detection limit of 0.27 pg for sodium sulfate levitated in an electrodynamic balance. Similarly, Haddrell et al. [24] used Raman spectroscopy to monitor an optically trapped droplet while it collected accumulation-mode aerosol particles by coalescence, demonstrating the detection of approximately 3 pg of accumulated aerosol material within 50 s. Optical trapping combined with Raman-spectroscopy measurements of single carbonaceous particles in air has been reported over the past 15 years. Pan et al. obtained Raman spectra from a single carbon nanotube particle trapped by the photophoretic force in air [25] and later developed the technology to measure temporally position-resolved Raman spectra from optically trapped single airborne particles [26]. Ling and Li [27] trapped and stabilized single absorbing carbonaceous particles with a focused laser beam to collect Raman spectra. Uraoka et al. [28] used an annular beam to levitate single light-absorbing carbonaceous particles and monitored chemical changes during heterogeneous oxidation. Gong et al. [29,30] used OT–RS to characterize and spatially resolve single airborne carbonaceous aerosol particles. Bergqvist et al. [31] demonstrated online Raman measurements for quantitative detection and characterization of aerosolized soot. Additionally, Ai et al. [32] applied OT and time-resolved Raman spectroscopy to measure bioaerosols, including pollen, bacteria, and fungi, and studied their atmospheric aging. These studies have well established the combination of OT and Raman spectroscopy as a feasible method for sensing and measuring single carbonaceous particles in air.

In this work, we apply an OT-RS platform for in-situ sensing of single, carbonaceous aerosol particles in freshly generated smoke. Smoke was produced by laser-induced ignition of nylon rope and plastic fibers, with the trapping laser also serving as the ignition source. Single particles were captured directly from the fresh smoke and immediately analyzed using Raman spectroscopy and imaging. This method minimizes sample contamination and the structural-property changes caused by collection. It also enables rapid sensing of single particles in their airborne environment, providing both physical and chemical information in their native state. Raman spectra from the two smoke sources are compared to identify their common and distinct chemical features. Finally, spatially resolved Raman measurements revealed compositional heterogeneity within individual micrometer-sized particles.

Note that the present study does not introduce a new trapping geometry or an enhanced detection limit. Instead, our focus centers on three key objectives: (1) in-situ sensing of freshly generated single carbonaceous aerosol particles while they remain in an airborne state; (2) rapid characterization of physical and chemical properties, providing the ability to differentiate between measured particles; and (3) high-spatial-resolution mapping to identify the compositional heterogeneity across an individual micrometer-scale particle. We demonstrate that all three core functionalities can be seamlessly integrated into a single system. These features represent fundamental and essential capabilities for a single-particle platform, paving the way for future on-site atmospheric aerosol sensing and monitoring.

## 2. Experiments

**Figure 1:**
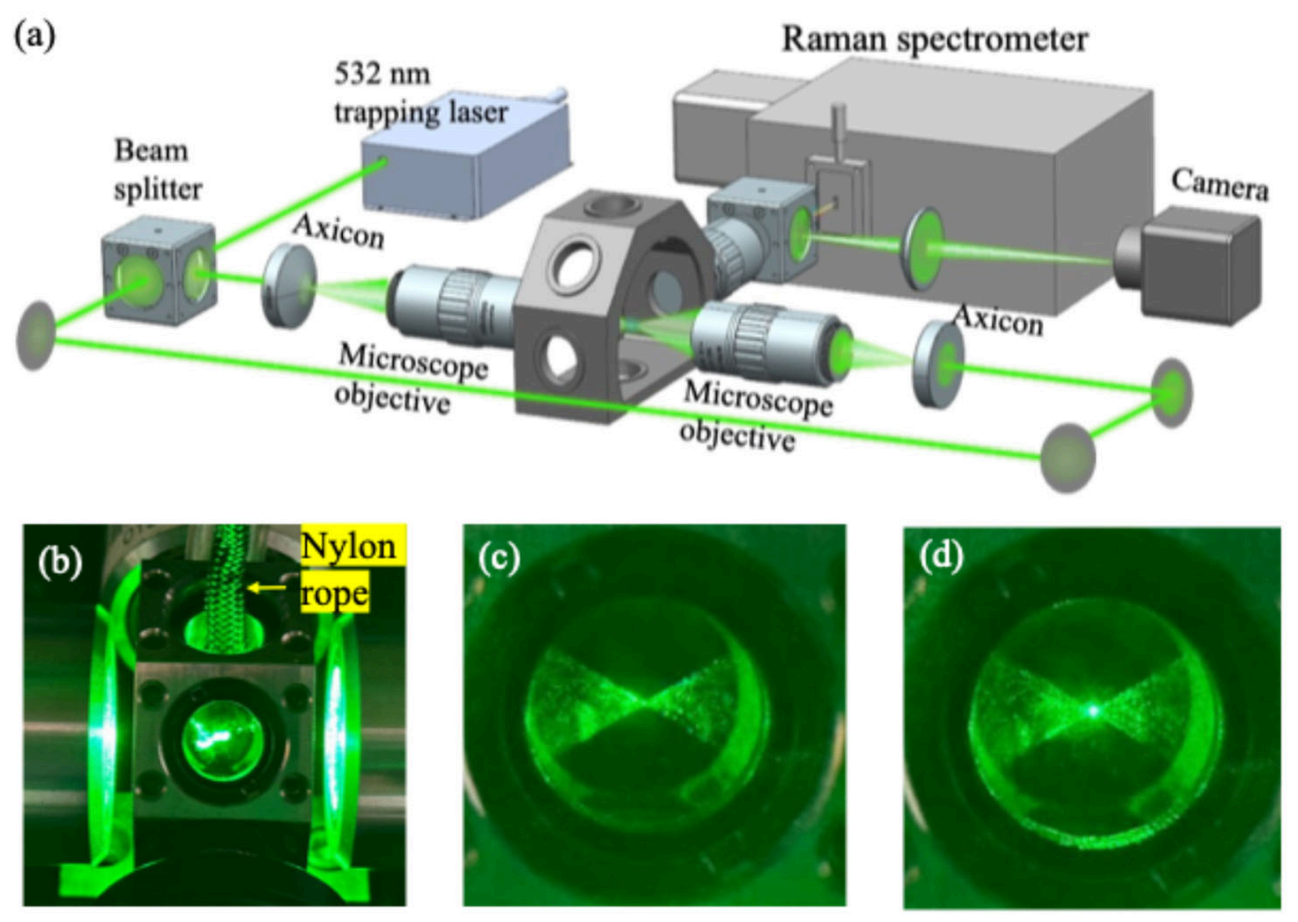
(**a**) Schematic of the OT–RS system for airborne single-particle sensing. A continuous-wave 532 nm laser beam is divided into two optical paths and converted into two counter-propagating hollow beams to form the UOT. Raman spectra and real-time images of the scattered light are recorded from the trapped particle. (**b**) Particle generation via the laser-induced ignition of a section of nylon rope that is inserted through the open top of the chamber. (**c**) Freshly generated smoke is illuminated by the counter-propagating hollow beams. (**d**) Image of a single particle from the smoke captured in the optical trap.

The OT-RS system [32,33], which combines a universal optical trap (UOT), Raman spectral recording, and optical imaging, is briefly described here and shown in Figure 1a. A continuous-wave 532 nm laser with a near-TEM_00_ beam profile is used for both particle trapping and Raman excitation. A 50:50 beam splitter divides the laser beam into two paths. Each beam passes through an axicon, which converts the Gaussian beam into a hollow beam. Two microscope objectives (N.A. = 0.55, Mitutoyo, Thorlabs, Newton, NJ, USA) focus the beams from opposite directions inside a chamber. The two counter-propagating hollow beams form the trapping region, known as the UOT. The trap size can be adjusted by changing the distance between the two objectives. Once a particle is captured by the UOT, Raman excitation is produced by the same 532 nm laser used for trapping. The scattered light is collected by a third objective (N.A. = 0.42, Mitutoyo, Thorlabs, Newton, NJ, USA) and divided into two portions by a dichroic beam splitter. The inelastically scattered light is directed into a spectrograph with a 600 grooves/mm grating for spectral dispersion and an Electron Multiplying Charge-Coupled Device (EMCCD) (Princeton Instruments, PROEM 1600 × 200, Teledyne Adimec, Billerica, MA, USA) for Raman spectral recording. An acquisition time of 30 s is commonly used for single particles in this study. The real-time particle image is recorded by a CMOS camera (Blackfly S, Teledyne Adimec, Billerica, MA, USA), which allows monitoring the particle’s physical information, including size, shape, and position. The imaging resolution is 0.18 ± 0.01 µm/pixel. Raman spectroscopy and real-time imaging are synchronized so that physical and chemical information is obtained from the same particle simultaneously.

In the experiment, the top window of the chamber is open to ambient air. A nylon rope or plastic fiber is inserted into the chamber through this open window and ignited by the laser beam, as shown in Figure 1b. The output laser power used for both ignition and trapping was 400 mW. Smoke was generated from a commercial nylon rope (polyamide 66) obtained from a local hardware store and an optical fiber (polystyrene-core, diameter of 500 μm, Edmund optics, Barrington, IL, USA) used for light transmission. After the smoke is generated, the sample is removed, while the chamber remains open to allow air exchange and maintain the atmospheric environment inside the chamber. Figure 1c shows the smoke illuminated by the counter-propagating hollow beams. This image was taken immediately after the nylon rope was removed from the chamber. Note that the smoke is distributed throughout the chamber, not only in the visible region. Only this region could be clearly observed because the smoke particles scattered light from the trapping beams. The particles moved randomly within the chamber. As a particle approached the trapping region, the optical force became stronger and guided it toward the center of the UOT, where it was trapped. Because the chamber is open, the pressure inside and outside the chamber is balanced with the surrounding atmosphere. Typically, a smoke particle can be captured by the UOT within 10 s after the sample is removed. Immediately after the particle is trapped, Raman spectroscopy and real-time imaging are conducted on the particle. The Raman acquisition time is 30s; therefore, each particle is trapped for approximately 30 s and then released by blocking the trapping beam. The UOT then captures another particle from the smoke. Normally, more than 10 particles can be trapped and characterized before the smoke is diluted by air exchange within the chamber. Figure 1d shows a single particle trapped in the optical trap. Raman spectra and real-time images are then recorded from the trapped particle.

In this study, “freshly generated smoke” refers to particles that remain airborne in the chamber after laser-induced ignition and are measured without a sampling process. Before trapping, the particle moves freely within the chamber without optical or manual transfer. Once trapped, it is measured directly at the trapping position. “In-situ” denotes the procedure in which particle trapping and measurement is conducted “on site”, enabling rapid characterization of freshly generated particles while they remained airborne in the chamber. Because particles were captured during the approximately 15–20 min period in which the smoke remains visible, the precise age of each particle varies at the time of capture. Therefore, the results do not suggest that every measured particle represents an unaltered primary emission. Physical and chemical transformations, including coagulation, oxidation, condensation, and other particle–particle interactions, may have occurred before a particle was trapped.

## 3. Results and Discussions

### 3.1. SEM Image and EDX Spectra of Collected Smoke Samples

**Figure 2:**
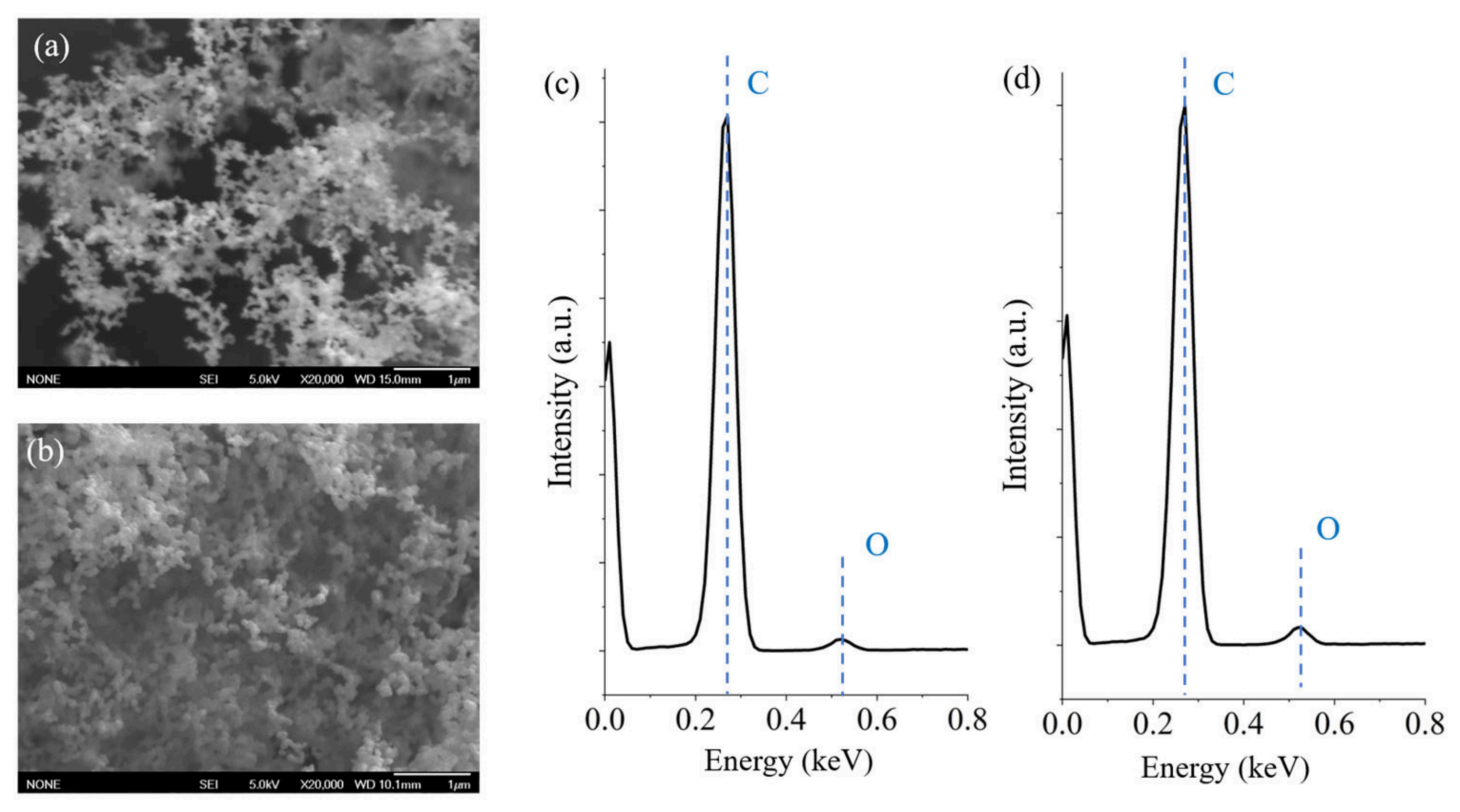
Morphology and elemental composition of collected smoke particles. (**a**,**b**) Scanning electron microscopy (SEM) images of particles generated by burning nylon rope and plastic fiber, respectively. (**c**,**d**) Corresponding energy-dispersive X-ray (EDX) spectra of these particles from nylon rope and plastic-fiber smoke, respectively.

To determine the size, shape, and elemental composition of the carbonaceous aerosol particles from the smoke, scanning electron microscopy (SEM) and energy-dispersive X-ray spectroscopy (EDX) analyses were conducted on the collected smoke particles. Figure 2a,b show SEM images of smoke particles from burning nylon and plastic fiber, respectively. The micrographs reveal that smoke from both sources forms irregularly shaped, micrometer-sized aggregates composed of much smaller particles. Both samples exhibit rough surfaces, irregular shapes, and distinct porosity, and the aggregates consist of numerous submicrometer-sized particles clustered together. However, a clear structural distinction is also observed: the plastic-fiber smoke forms larger, more rounded, and more compact aggregates, whereas the nylon smoke has a more open and chain-like structure.

Figure 2c,d show the EDX spectra of smoke particles produced by burning nylon rope and plastic fiber, respectively. Because hydrogen cannot be detected by EDX, carbon and oxygen constitute the primary identified elements. These results indicate that carbon is the dominant element in both samples, confirming the presence of oxygenated carbonaceous material.

The particles measured by SEM/EDX were collected on carbon tape and were not the same single particles measured by OT–RS. These results provide complementary information about the collected particles from each smoke source, rather than a direct measurement of the morphology or elemental composition of the optically trapped particles. Deposition may flatten or rearrange the shape, and the carbon tape may contribute to the EDX carbon signal. Therefore, SEM/EDX results are used only as supporting information and do not independently confirm the Raman-band assignments or the composition of each trapped particle. The contact-free characterization of airborne particles applies to the OT–RS measurements.

### 3.2. Single-Particle Raman Spectra and Real-Time Images of Particles from Smoke

**Figure 3:**
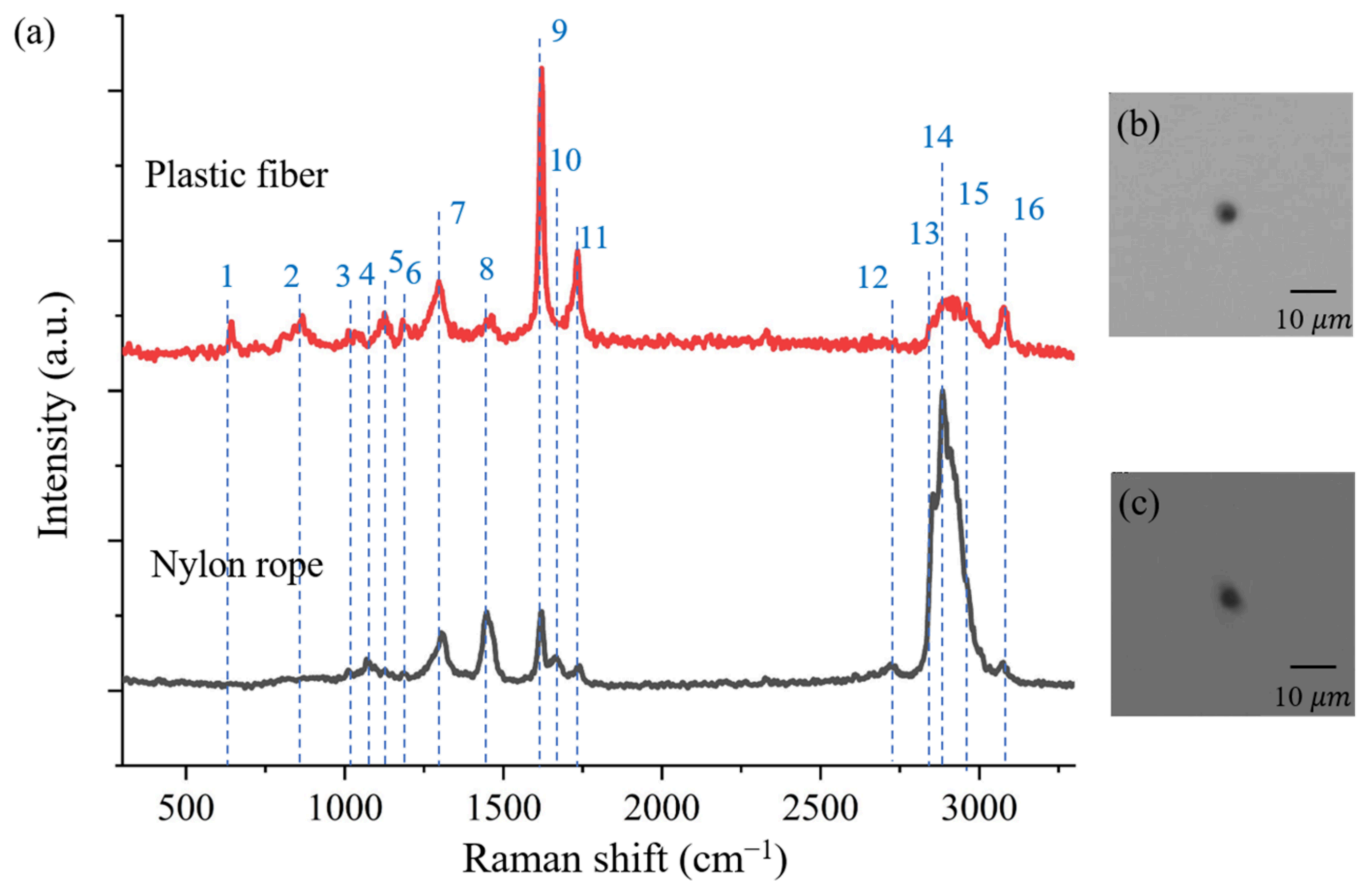
(**a**) Raman spectra of optically trapped single carbonaceous particles from plastic-fiber and nylon-rope smoke. The spectra contain both common bands and distinct bands, providing characteristic spectral fingerprints that differentiate the measured particles from the two smoke sources. The numbers 1–16 are the Raman peak numbers. Detailed Raman-band assignments and comparisons are provided in Table 1. (**b**,**c**) Real-time images of the corresponding trapped particles from plastic-fiber and nylon-rope smoke, respectively. The estimated spatial dimension of the trapped particles is approximately 3.0 μm across.

The single-particle Raman spectra of smoke particles produced by burning nylon rope and plastic fiber are shown in Figure 3a, and the corresponding band assignments are shown in Table 1. More than 100 single particles were measured for each smoke type. The main Raman-band positions and characteristic spectral features were reproducible among particles from the same smoke source. The spectra shown here are representative examples of the spectral features. The presence of shared Raman bands indicates that both particle types possess similar functional groups. Specifically, a band near 1080 cm^−1^ arises mainly from C-C skeletal stretching [34,35]. The band near 1450 cm^−1^ is assigned to CH_2_ and CH_3_ deformation, indicating the presence of aliphatic hydrocarbon groups [34,35,36]. Features in the 1610 cm^−1^ region contain contributions from conjugated or aromatic C=C structures. The band near 1740 cm^−1^ corresponds to carbonyl C=O stretching and indicates oxygen-containing compounds such as ketones, aldehydes, carboxylic acids, or esters [34,35,36]. Lastly, the broad band between approximately 2700 and 3000 cm^−1^ is produced by aliphatic C-H stretching, verifying the organic nature of both smoke samples [34,35,36,37].

Several distinct spectral differences were also observed. The particle from plastic-fiber smoke shows bands near 630 and 850 cm^−1^. These bands are consistent with aromatic-ring deformation and aromatic-ring C-C stretching, respectively [35]. These assignments suggest the presence of aromatic structures in the plastic-fiber smoke particle, consistent with the aromatic rings in the polystyrene source material. Such structures may be retained or formed during combustion and contribute to the spectral differences between plastic-fiber and nylon-rope smoke particles. Conversely, the particles from nylon smoke exhibit a distinct band near 1660 cm^−1^ [34,35] corresponding to the amide I vibration. This suggests that part of the original polyamide structure may remain in the smoke. Because nylon contains nitrogen in its polyamide backbone (–CO–NH–), nitrogen in freshly generated smoke may originate from the original material [38]. Based on the Raman-band assignments, a large proportion of the particles present in the smoke appear to be unburned. However, during combustion, most fuel-bound nitrogen is released into the gas phase as HCN, NH_3_, NO, or NO_2_ [38], leaving only a small fraction in the condensed particle. Furthermore, NO_2_ may also react with soot or OC to form nitrogen-containing secondary products [39] via a rapid aging pathway occurring immediately between emitted gases and particles in the smoke. Notably, as shown in Figure 2d, the EDX spectrum lacks a detectable nitrogen signal. In this case, the band may not originate from the unburned source material; instead, it may be associated with a small amount of secondary organic aerosol. Because this component represents only a minor fraction of the total mass, its elemental signal may remain below the detection threshold of bulk measurements like EDX. Nevertheless, particles from the two smoke sources can be unambiguously distinguished based on their unique Raman signatures.

The insets in Figure 3b,c present real-time images of the trapped particles. Particle size in this study refers to the spatial dimension measured from the real-time images. The OT–RS system typically traps particles ranging from several to several tens of micrometers, depending on particle properties and trapping conditions. The smallest particle successfully trapped in our experiments was a standard silica bead with a diameter of 2 μm (Whitehouse Scientific Ltd., Waverton, UK). Nanoscale particles can also be trapped, but they will form clusters with micrometer-scale dimensions. In this study, “single particle” refers to one object held and measured in the optical trap. This object may be a cluster formed from smaller particles, but the cluster is treated as one single particle during OT–RS measurement. Assuming the particle has a spherical morphology, its spatial extent of approximately 3.0 μm would correspond to a volume of 14.1 μm^3^. Compared with the SEM images, the trapped particle appears to be a complex, fluffy cluster formed by numerous smaller primary particles. Because these clusters contain both OC and EC, their effective density is highly variable. Large soot clusters (>300 nm) can have densities below 0.3 g/cm^3^ [40], while the reported density of OC ranges from 0.27 to 1.94 g/cm^3^ [41,42]. Based on the reported density range, the estimated mass of the trapped particle is approximately 3.8–27.4 pg. This result indicates that an exceptionally small sample mass on the order of 10 pg is sufficient to extract detailed chemical and structural information from a single carbonaceous particle using the OT-RS system. Different measured particles can also be clearly distinguished based on their Raman signatures. Note that the particle mass was estimated from a spherical approximation, giving a mass range of 3.8–27.4 pg. This is an assumption-based estimate rather than a directly measured mass. The estimate indicates an approximate sample amount of several to several tens of picograms under the stated assumptions. It also does not represent the Raman detection limit or the minimum particle size that the system can trap.

### 3.3. Spatially Resolved Raman Spectra of Single Particles from Smoke

**Figure 4:**
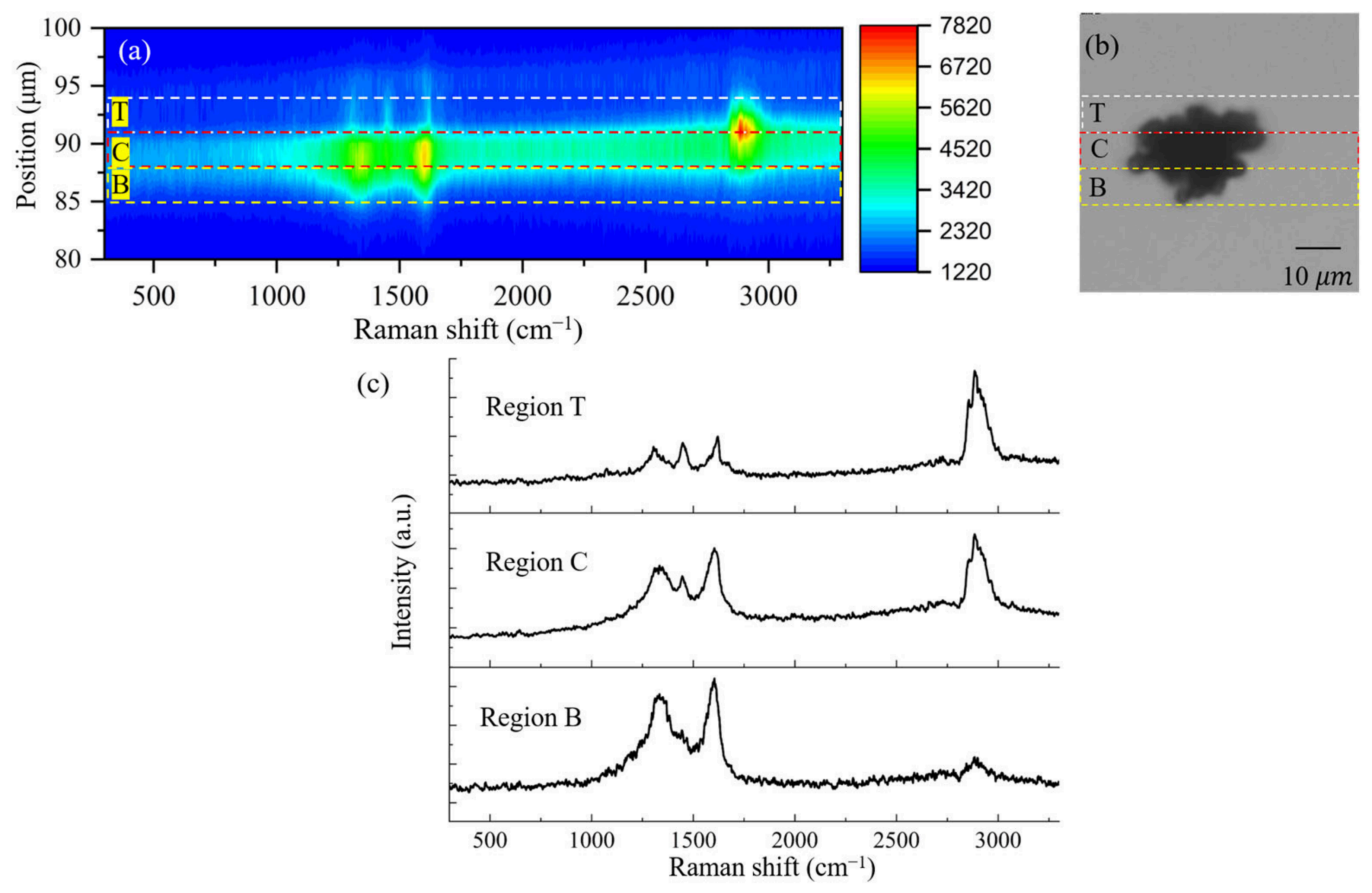
Spatially resolved Raman characterization of a trapped single particle from nylon smoke. (**a**) Contour plot showing the spatial distribution of Raman spectral intensity across the particle with the top (T), center (C), and bottom (B) measurement locations marked. (**b**) Real-time image of the trapped particle. (**c**) Position-resolved Raman spectra collected at T, C, and B, illuminating variations in chemical composition.

The smoke particles analyzed in this study contain both OC and soot. Some particles may contain a mixture of these components and are therefore chemically heterogeneous. To examine intraparticle variation, Raman spectra were collected at three locations on a trapped large smoke cluster: T (top), C (center), and B (bottom). Figure 4a shows a contour visualization of the spectral intensity, Figure 4b shows the real-time particle image, and Figure 4c displays the three position-resolved spectra. The integration time for Figure 4a is also 30 s. As shown in Figure 4a, the Raman intensity varies across a vertical distance of approximately 20 μm, which agrees with the particle’s vertical dimension observed in Figure 4b. The 20 μm span of the particle was divided into three measurement regions (T, C, and B), with each region covering approximately 7 μm.

The Raman spectrum position T (top) shows spectral features similar to those in Figure 4a. The bands at approximately 1305 cm^−1^, 1450 cm^−1^, 1610 cm^−1^, and 2700–3000 cm^−1^ are assigned to C-C/C-O-C stretching, C-H deformation, C=O stretching, and C-H stretching, respectively [34,35,36]. These features indicate that the upper part of the cluster is rich in OC. At position B (bottom), the Raman bands of OC are very weak. Instead, two clear bands are observed at approximately 1350 and 1590 cm^−1^. The peak at 1350 cm^−1^ is the D band, which is related to defects and disorder in sp^2^-bonded carbon [43,44]. The 1590 cm^−1^ peak is the G band, which arises from the stretching of sp^2^-bonded carbon atoms [43,44]. The two bands are characteristic of disordered graphitic carbon in soot, indicating that the lower part of the cluster is soot-rich. Furthermore, the small hump near 2900 cm^−1^ may arise from the D + G band of disordered carbon, further supporting the interpretation that soot is the main component [43,44]. At position C (center), Raman bands from both OC and soot are observed, although some bands overlap. Collectively, these spatial variations reveal that the trapped particle is a complex, heterogeneous mixture wherein OC is predominantly localized in the upper region and soot is concentrated in the lower region, with an uneven distribution.

The Raman image in Figure 4a appears slightly tilted because of the alignment of the EMCCD. This tilt does not affect the Raman-peak positions but may slightly affect the spatial regions measured at different heights. Nevertheless, it does not change the main conclusion regarding the surface chemical heterogeneity discussed in this section. The distinct Raman features observed across these regions demonstrate that the OT–RS system can resolve spatial differences within a single trapped particle. However, 7 μm represents the sampling scale used in this measurement and does not represent the spatial-resolution limit of the system. The actual spatial resolution depends mainly on the excitation wavelength, the numerical aperture of the microscope, and the resolution of the spectrometer and the recording EMCCD. The OT-RS configuration used in the current work has a spatial resolution of approximately 600 nm × 600 nm [45].

Carbonaceous aerosol particles are complex and often comprise diverse carbon-bonding structures, functional groups, coatings, and mixed components. This section showcases that single-particle analysis using spatially resolved OT–RS can not only rapidly characterize physical and chemical properties, but also reveals spatial differences in composition across a single carbonaceous particle at the micrometer scale. These capabilities are crucial for on-site field aerosol studies because they provide insights that extend beyond the general classification of BC and BrC or OC and EC. Furthermore, they enable the measurement of particles in their native, airborne state without requiring sample collection. Such combined capabilities remain limited in current commercial aerosol instruments.

## 4. Conclusions

In this work, OT–RS was applied for in-situ sensing of single carbonaceous particles from freshly generated smoke. Smoke particles from burning nylon rope and plastic fiber are directly trapped in air and immediately analyzed using Raman spectroscopy and real-time imaging to achieve in-situ measurement. This is a contact-free method of sample collection, thereby reducing sample contamination and enabling rapid characterization of individual single particles in their airborne environment, providing information that is closer to their native state. Although the measured particles had an estimated mass of the order of only 10 pg, this small amount is sufficient to generate detailed Raman spectra with clear chemical information, revealing both similarities and distinct differences between the particles from two smoke sources. In comparison, the particles from plastic-fiber smoke show unique bands near 630 cm^−1^ and 850 cm^−1^ related to aromatic-ring deformation and aromatic-ring C-C stretching. These distinct Raman spectral features indicate that OT–RS can effectively differentiate aerosol particles from their smoke emission sources. Furthermore, the spatially resolved Raman spectra revealed that the trapped cluster particles are chemically heterogenous. The upper region is rich in OC, as shown by its clear C-C, C=O, and C-H bands. The lower region is rich in soot-like carbon, as shown by the D and G bands near 1350 cm^−1^ and 1590 cm^−1^. These results confirm that OC and soot can phase-segregate into distinct regions within a single, airborne particle.

Overall, these results demonstrate that OT–RS enables in-situ, contact-free sensing of single, airborne particles, characterizing size, composition, and spatial heterogeneity using substrate-induced artifacts with only a minimal amount of material in their fresh native state. This minimizes potential alterations in particle surface properties, loss of volatile or semi-volatile components, contamination, and other sampling artifacts that can occur during conventional offline and bulk measurements, thereby providing information more representative of freshly generated airborne particles. The present study highlights three key sensing capabilities: (1) in-situ, contact-free characterization of freshly generated carbonaceous aerosol particles while they remain airborne; (2) simultaneous measurement of physical and chemical properties of individual particles, enabling particles from different smoke sources to be differentiated based on their Raman features; and (3) spatially resolved characterization of chemical heterogeneity within a single particle. Importantly, these capabilities are integrated within a single measurement platform and provide information that can be obscured by ensemble averaging in bulk measurements. These capabilities provide a foundation for future in-situ, single-airborne-particle sensing and monitoring.

The present study used particles from only two smoke sources to demonstrate the capability of OT–RS. Although the results show that the system can distinguish the measured particles, they do not quantitatively present its ability to identify airborne carbonaceous particles in complex combustion environments, such as wildfires. In this work, the particle size was estimated, introducing uncertainty into the calculated mass. More precise characterization would require additional optical components, such as an extra CCD to record elastic-scattering information for size estimation using Mie theory. A laser with a different wavelength and a photomultiplier tube (PMT) could also support absorption measurements and help estimate particle density. Therefore, future work will include measuring particles from a wider range of smoke sources and adding these optical components to the system. These developments will improve particle characterization and help assess the system’s suitability for on-site sensing in complex environments, including wildfire smoke.

## Figures and Tables

**Table 1:** Assignments of the measured Raman bands from the optically trapped single particles.

Peak Numbers	Bands (cm^−1^)	Assignments	Reference
1	630	aromatic-ring deformation	[35]
2	850	aromatic-ring C-C stretching	[36]
3	1057	C-O stretching	[34]
4	1080	C-O-C stretching glycosidic link, C-C skeletal	[34]
5	1176	C-H bending	[35]
6	1205	C-C stretching	[36]
7	1305	CH_2_ deformation	[35]
8	1450	C-H vibration, CH_2_ bending, CH_2_CH_3_ deformation	[34,35]
9	1610	C=C stretching	[36]
10	1660	Amide I vibration mode or nitrogen-bond reaction products	[34,35]
11	1740	carbonyl C=O stretching	[36]
12	2725	CH stretching vibration	[35,37]
13	2890	CH_2_ asymmetric stretch	[35,37]
14	2922	CH_2_ stretch	[35,37]
15	2975	CH_2_ stretch	[35,37]
16	3100	=C-H stretching	[35,37]

## Data Availability

The data supporting the findings of this study are included in the article. Additional raw Raman spectra, SEM/EDX data, and particle images are available from the corresponding author upon reasonable request.
